# Quality blues: traditional knowledge used for natural indigo identification in southern China

**DOI:** 10.1186/s13002-021-00454-z

**Published:** 2021-04-07

**Authors:** Yuru Shi, Libin Zhang, Lu Wang, Shan Li, Zuchuan Qiu, Xiaoyong Ding, Yuhua Wang

**Affiliations:** 1 Department of Economic Plants and Biotechnology, Yunnan Key Laboratory for Wild Plant Resources, Kunming Institute of Botany, Chinese Academy of Sciences, 132# Lanhei Road, Kunming, 650201 China; 2 University of Chinese Academy of Sciences, Beijing, 100049 China; 3 Key Laboratory for Microbial Resources of the Ministry of Education, Yunnan Institute of Microbiology, School of Life Sciences, Yunnan University, Kunming, 650091 China

**Keywords:** Ethnobotanical survey, Indigo paste, Folk quality criteria, Quantitative study, Indirubin, Traditional knowledge, World heritage

## Abstract

**Background:**

As one of the oldest traditional dyes, people worldwide have used natural indigo for centuries. Local people have unique knowledge about indigo identification, which is crucial for indigo quality control and determining the dyeing effects. However, such traditional knowledge is rarely documented and explained. Therefore, the aims of this study were to document and assess the traditional knowledge used by local people when identifying natural indigo paste as well as quantitatively explore the characteristics and material basis of such traditional knowledge.

**Method:**

Three field surveys were conducted between 2019 and 2020. A total of 283 traditional indigo-paste artisans were interviewed in Guizhou, Yunnan, and Fujian Provinces. The frequency of citation, mention index, and fidelity level of each indigo-paste quality criterion were calculated to determine the most commonly used, recognized, and important quality criteria. To explore the characteristics and material basis of the traditional knowledge, we analyzed 21 indigo-paste samples using high-performance liquid chromatography with diode-array detection (HPLC-DAD), pH, and particle size analyses.

**Results:**

Local people possess unique knowledge to identify natural indigo. Based on this knowledge accumulated over thousands of years, four criteria (color, taste, touch, and dyeing ability) were chosen by local people, and using these criteria, nature indigo was divided into five quality grades. The best quality indigo paste was judged according to the following folk criteria: dark blue in color with a purple-red luster; smooth and difficult to wipe off; having a sweet, bitter or spicy taste; and easy cloth dyeing. Additionally, the higher the contents of indigo and indirubin—especially indirubin—the better is the quality of the indigo paste. Within the pH range of 9–12, high-quality indigo-paste was more acidic. There was no significant relationship between particle size and quality.

**Conclusion:**

The ancient methods used by local people for identifying natural indigo are comprehensive and unique. By documenting the various folk quality criteria and conducting quantitative analyses, this study revealed the importance of indirubin and pH for assessing the quality of indigo paste. These findings differ from existing quality standards for synthetic indigo. Amid rapid modernization, traditional knowledge remains invaluable as a world heritage of humanity that warrants preservation.

## Background

Natural indigo is considered one of the oldest dyestuffs known to humanity, and one of the most commonly used natural dyestuffs worldwide, particularly in Egypt, India, China, and Africa [1, 2]. It is extracted from indigo-yielding plant species. To date, 31 plant species across eight different families have been used as sources of indigo (e.g., *Indigofera tinctoria* L. and *I. suffruticosa* Mill.) [3]. Indigo-yielding plant species are processed using a variety of methods developed in different regions, such as *sukumo* (*Persicaria tinctoria* [Aiton] Spach) in Japan and woad balls (*Isatis tinctoria* L.) in Europe. Indigo paste is traditionally used in China. Over thousands of years, natural indigo has been integrated into the cultures of various ethnic groups and used in ethnically characteristic ways, including tie-dyeing of the Bai people, batik of the Miao people, and in the production of the bright cloth of the Dong people [4, 5]. Further, the color of indigo has been endowed with a unique significance and is commonly used as a symbol of independence and individualism, whereby it is known as *the king of colors* and *the color of kings* [6]. However, with the rise of industrialization, synthetic indigo is now used in almost the entire dyeing industry, owing to advantages such as high purity, low price, and better producibility [7–10]. Therefore, the production and use of natural indigo has declined and gradually disappeared such that it currently occurs only in a few remote areas in China, India, and a few other countries [11, 12]. However, the disadvantages of using synthetic dyes include the high costs of the raw materials, their toxicity, and environmental pollution [7, 8, 13–17]. Therefore, in recent years, consumer interest in natural indigo has been on the rise, which has attracted the attention of dyeing enterprises.

In contrast with synthetic indigo, natural indigo is considered a *green* dye that has a harmonious and sustainable relationship with the environment, as it is biodegradable [11, 15, 18]. In terms of dyeing, natural indigo has many advantages over synthetic indigo. For example, synthetic indigo contains only residual chemical impurities apart from the indigo component, whereas natural indigo is a mixture containing 7–45% indigo, in addition to indirubin, dark brown indigo, and yellow indigo [1, 19]. These components give fabrics dyed with natural indigo a richer color and better colorfastness than fabrics dyed with synthetic indigo [1, 20, 21]. In addition to its pleasant natural fragrance, fabrics dyed with natural indigo have certain health benefits, as they show insect-repellent and disinfectant properties [18, 22].

Unfortunately, the quality of natural indigo currently found in the market varies considerably, and to date, there is no reliable method for evaluating the quality of this natural dye. However, indigo identification significantly influences indigo quality control and the determination of its dyeing effects [23–26]. While numerous studies on indigo-yielding plant species and traditional indigo extraction and dyeing methods have been conducted, very little is known about the folk criteria used for indigo identification.

Previously, we found that people still cultivate, use, and trade natural indigo (as indigo paste) on a large scale, in parts of Guizhou, Yunnan, and Fujian Provinces in southern China. Further, we have learned that traditional knowledge about indigo paste is passed on from generation to generation and that local people separate indigo paste into different quality grades using folk criteria. However, such traditional knowledge has rarely been documented and explained. In light of this, the present study aimed to document and assess the traditional knowledge of the local people in identifying the natural indigo paste. Further, it sought to as well as quantitatively explore the characteristics and material basis of such traditional knowledge.

## Methods

### Study sites

**Fig. 1:**
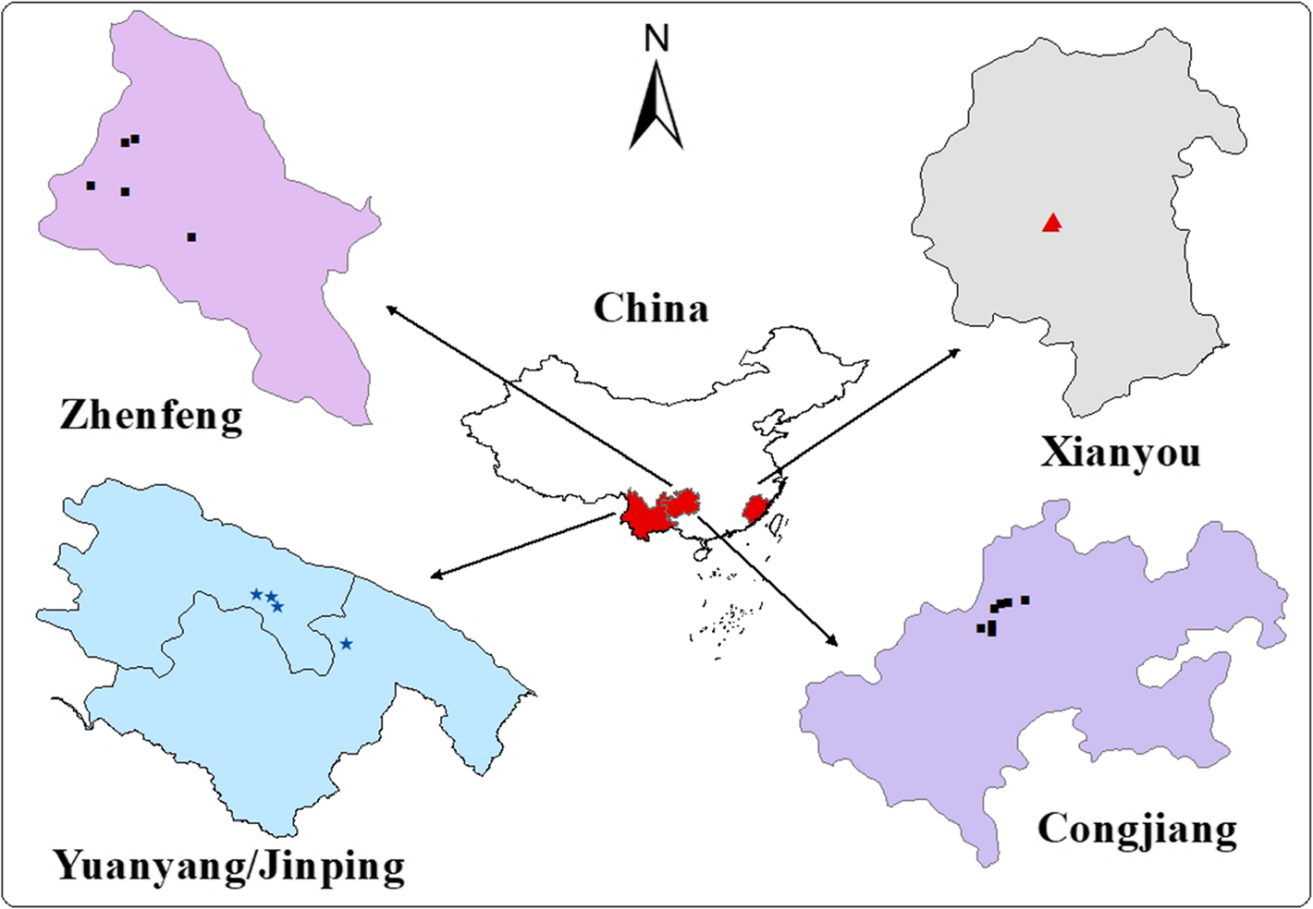
Three markets and 15 villages in Guizhou, Yunnan and Fujian were selected as the survey sites

Information provided by the local governments, together with a preliminary survey, played a decisive role in selecting the research sites. We identified 15 villages and 3 markets in Guizhou, Yunnan, and Fujian Provinces as suitable survey sites because people in these areas frequently used indigo paste and had a good heritage of traditional knowledge. Guizhou and Yunnan Provinces are located in Southwest China, whereas Fujian Province is located in Southeast China (Fig. 1).

Congjiang County (25°16′–26°05′ N; 108°05′–109°12′ E) and Zhenfeng County (25°07′–25°44′ N; 105°25′–105°56′ E) [27] belong to the Qiandongnan Miao and Dong Autonomous Prefecture and Qianxinan Buyi and Miao Autonomous Prefecture, respectively. Congjiang County is located in the middle reaches of the Duliu River. The climate is classified as humid monsoon of the mid-subtropical zone. The annual average temperature and precipitation are 18.5 °C and 1185.9 mm, respectively. Ninety-five percent of the population in Congjiang County is composed of ethnic minorities, such as the Miao and Dong peoples [28], providing an ideal environment for this study because of their strong cultural inheritance practices. The six villages surveyed in Congjiang County were located in mountainous areas on both sides of the Duliu River, and the residents were all Miao people. Currently, the local area retains a strong indigo-paste culture, and the daily dress of Miao women is still the traditional national costume. Every family in the local area cultivates the indigo-yielding plant, *Strobilanthes cusia* (Nees) Kuntze, on an annual basis to prepare indigo paste for dyeing cloth. Zhenfeng County has a subtropical humid monsoon climate with an annual average temperature and precipitation of 16.6 °C and 1276.9 mm, respectively [27]. In Zhenfeng County, indigo paste is traded as a commodity and is thus produced on a large scale. The local trade in indigo paste takes place on market day at the farmers market in each town. Sellers are mostly Han residents from nearby villages and buyers are local ethnic minorities and merchants. In Zhenfeng County, we conducted surveys at the two main farmers markets and in the three villages that produce indigo paste.

Yuanyang County (22°49′–23°19′ N; 102°27′–103°13′ E) and Jinping County are located in the Honghe Hani and Yi Autonomous Prefecture of Yunnan Province. Yuanyang County has a subtropical mountain monsoon climate with an annual average temperature and rainfall of 24.4 °C and 899.5 mm, respectively. Yuanyang County is inhabited by seven ethnic groups including the Hani, Yi, and Han peoples, who have lived in this area for generations. Ethnic minorities account for 89.44% of the total population [29]. Jinping County has a tropical monsoon climate with an annual average temperature and rainfall of 18 °C and 2358.6 mm, respectively. Nine ethnic groups, including the Miao and Yao peoples, have lived in the area for generations and ethnic minorities account for 87.6% of the total population [30]. In the four villages surveyed in the Yunnan Province (one Yao and three Hani villages), the elderly retain and practice traditional natural-indigo culture and artisanship.

Xianyou County (25°11′–25°43′ N; 118°27′–118°56′ E) is located approximately halfway along the coastline of Fujian Province, across the island of Taiwan. The climate is classified as south subtropical maritime monsoon, with an annual average temperature of 20.6 °C and total rainfall ranging from 300 to 2300 mm. In this county, the town of Shufeng is reputedly *the Hometown of Indigo Naturalis*, because of its long history of making high-quality Indigo Naturalis [31], the powder processed from indigo paste. Its active ingredients are indigo and indirubin, and it is used medicinally to treat oral ulcers [32], ulcerative colitis [33], and psoriasis [34–36].

### Field survey and data collection

The first field survey was conducted between August and September 2019, over approximately 10 days in each of Congjiang and Zhenfeng Counties of Guizhou Province; YRS, ZCQ, and XYD conducted the field surveys and collected the ethnobotanical data and voucher samples. The second field survey was conducted over 7 days in October 2019 in Xianyou County of Fujian Province; YRS and LW carried out the field surveys and collected the ethnobotanical data. The third field survey was conducted over 6 days in January 2020 in Yuanyang and Jinping Counties of Yunnan Province; YRS, LBZ, and LW carried out the field surveys and collected the ethnobotanical data. During the field investigation, we invited local people who could speak the local language and Mandarin to serve as interpreters. Methods used for data collection included, purposive sampling [37], snowball sampling [38], participatory observation, and a questionnaire survey [39]. The interview questionnaire is shown in Table 1. All interviewees possessed traditional knowledge related to indigo paste. Informed consent from all interviewees was obtained orally before conducting the interviews. Once permission had been obtained, we captured photographs [40], created audio and video recordings, and collected other materials to assist our research.

**Table 1:** Questionnaire for the interviewees.

1 How many indigo-yielding plant species do you use?	
2 What are the local names of these indigo-yielding plants?	
3 What do these local names mean?	
4 How to make indigo paste after harvesting indigo-yielding plants?	
5 How many ways can you judge the quality of indigo paste?	
6 How to judge specifically?	
7 Which of these methods do you like best?	

As shown in Table 2, a total of 283 informants were interviewed, including 171 from Guizhou Province (139 from Congjiang County and 32 from Zhenfeng County), 42 from Fujian Province, and 70 from Yunnan Province. The age of the interviewees ranged between 31 and 81 years, with 88.0% ranging between 30 and 69 years. The number of female interviewees (*n* = 219) was almost 3.5 times that of the male interviewees (*n* = 64).

**Table 2:** Sex and age of the interviewees

	Number	Percentage
**All**		
Sex		
Male	64	22.6%
Female	219	77.4%
Age		
30–49	101	35.7%
50–69	148	52.3%
≥ 70	34	12.0%
**Guizhou**		
Sex		
Male	26 (C:0, Z:26)	15.2% (C:0%, Z:81.3%)
Female	145 (C:139, Z:6)	84.8% (C:100%, Z:18.7%)
Age		
30–49	79 (C:70, Z:9)	46.2% (C:50.4%, Z:28.1%)
50–69	78 (C:56, Z:22)	45.6% (C:40.3%, Z:68.8%)
≥ 70	14 (C:13, Z:1)	8.2% (C:9.3%, Z:3.1%)
**Yunnan**		
Sex		
Male	8	11.4%
Female	62	88.6%
Age		
30–49	18	25.7%
50–69	38	54.3%
≥ 70	14	20.0%
**Fujian**		
Sex		
Male	30	71.4%
Female	12	28.6%
Age		
30–49	4	9.5%
50–69	32	76.2%
≥ 70	6	14.3%

Note: *C* Congjiang County, *Z* Zhenfeng County

### Quantitative analysis of the ethnobotanical data

To screen out the most commonly used, most recognized, and most important quality criteria, we used questions 5, 6, and 7 (Table 1) to calculate the Frequency of Citation (FC), Mention Index (QI) [41], and Fidelity Level (FL) [42] of each quality criterion, respectively. The number of interviewees using each quality criterion was counted as the FC for the criterion. QI, used to test knowledge homogeneity, was calculated using the following formula: QI = number of mentions/number of interviewees. In turn, FL, used to evaluate the importance of the different quality criteria, was calculated using the following formula: FL = (total number of interviewees providing one quality citation/total number of interviewees providing all quality criteria) × 100%.

### Chemical analysis

To avoid sample interference, we obtained 21 indigo-paste samples (all extracted from *Strobilanthes cusia*) from Guizhou Province, which had the largest number of indigo-paste users among all study sites. These 21 indigo-paste samples were identified and categorized by 3 key informants using 5 quality levels: best (3 samples), good (3 samples), general (7 samples), poor (5 samples), and worst (3 samples). We used the values 1, 2, 3, 4, and 5 to represent the 5 quality grades of indigo paste, respectively.

An Agilent 1260 series equipment (Agilent Technologies, USA) was used to quantitatively analyze the active ingredients (i.e., indigo and indirubin) present in the indigo-paste samples [3]. Ground indigo powder samples (0.5 g; measured to 0.0004 g accuracy) were ultrasonicated for 30 min to completely disperse the dye in 50 ml of distilled water at pH 7 [43]. A PHS-3C acidity meter and E-201-C composite electrode (Shanghai INESA Scientific Instrument Co. Ltd., Shanghai, China) were used to measure the pH of the 21 indigo-paste samples. An indigo-paste suspension (0.1 g/L) was prepared using distilled water, and the upper part of the suspension was withdrawn for particle size analysis after ultrasonic dispersion for 10 min. A Malvern Zetasizer ZEN 3600 zeta potential analyzer (Malvern Instruments Ltd., Malvern, U.K.) was used for particle size testing [44].

### Statistical analysis

Analysis of variance (ANOVA) was conducted to determine any significant effects of active ingredient content and pH (*p* ≤ 0.05) on the quality grade of the indigo-paste samples. Differences in particle size of the different quality grades of indigo paste were analyzed using the Origin Pro learning edition data-analysis software to produce a line graph of the particle size distribution of the 21 indigo-paste samples for comparison.

## Results

### Traditional knowledge used for indigo identification

**Fig. 2:**
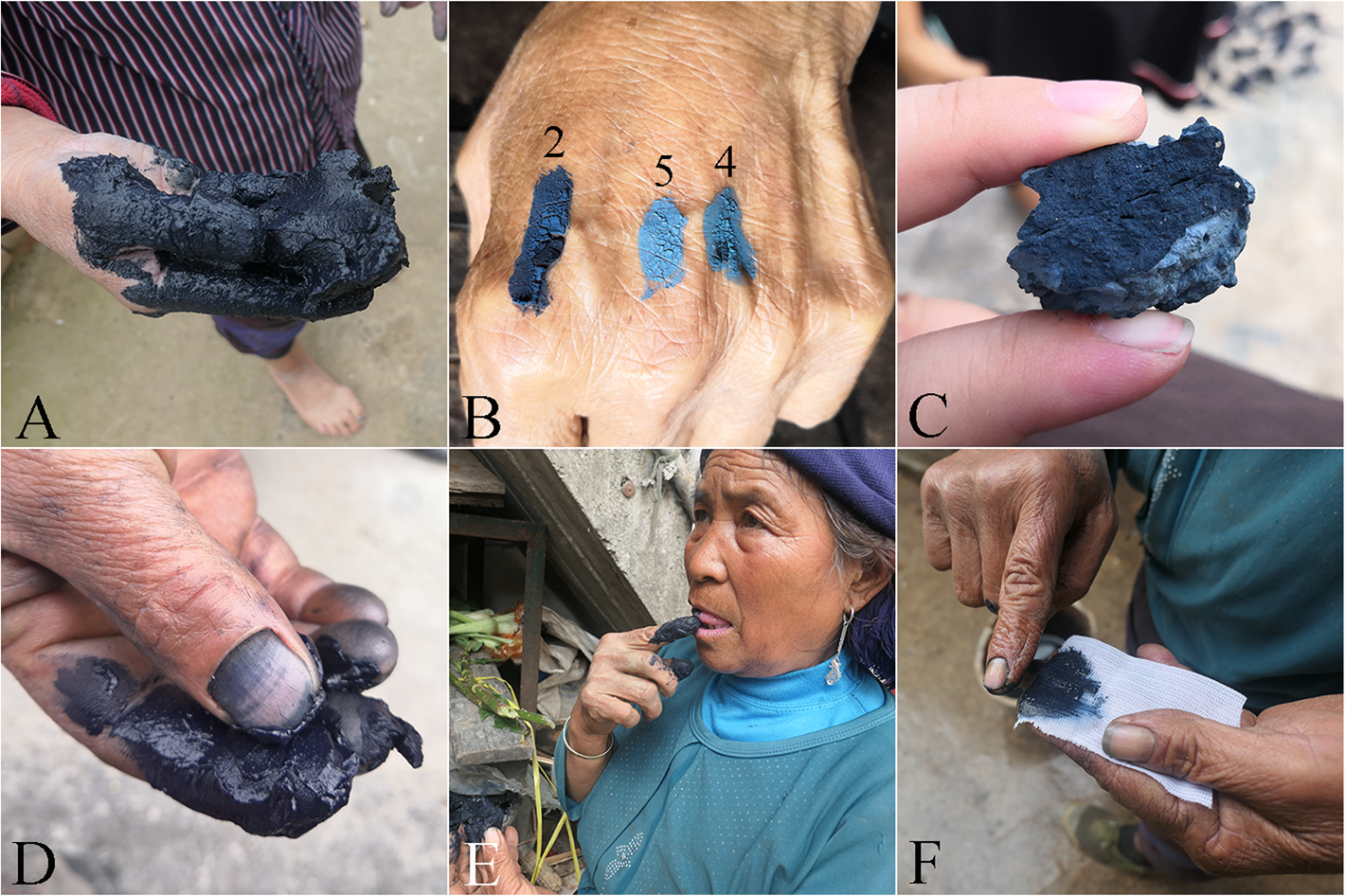
Different folk quality criteria. **a**–**c** Method of color criterion. **d** Method of touch criterion. **e** Method of taste criterion. **f** Method of dyeing ability criterion. 1–5 represent the five folk quality grades of indigo paste, which are best, good, general, poor, and worst respectively

As shown in Table 3, five quality grades and four quality criteria for indigo paste were documented. The five quality grades were best, good, general, poor, and worst, while the four quality criteria were color, taste, touch, and dyeing ability (Fig. 2). All four of these criteria were used in Yunnan and Guizhou Provinces, whereas only color and touch were used in Fujian Province.

**Table 3:** Quality criteria used by folk to assess indigo paste.

Criteria	Folk quality levels					Guizhou	Yunnan	Fujian
	1	2	3	4	5	FC/QI/FL	FC/QI/FL	FC/QI/FL
Color	Dark blue, deep purple-red	Dark blue, reddish	Blue	Blue-black, black	Light blue, bluish grey, turquoise	171/1.00/100%	70/1.00/100%	42/1.00/100%
Touch	Exquisite and smooth	Exquisite	Slight granular sensation	Granular sensation	Obvious granular sensation	20/0.12/12%	5/0.07/7%	9/0.21/21%
Taste	–	–	–	–	–	47/0.27/27%	2/0.03/3%	0/0.00/0%
Dyeing ability	Easy	Easy	General	Difficult	Hard	11/0.06/6%	3/0.04/4%	0/0.00/0%

Note: – meaning that the folk description of this criterion in disagreement

In Congjiang County of Guizhou Province and Yunnan Province, people grade indigo paste only in its wet state, and they believe that high-quality indigo paste should have dark-blue hue and purple-red metallic luster (Fig. 2a). However, in Zhenfeng County of Guizhou Province, people also assessed the indigo paste in its dry state by applying wet indigo paste to a small area on the back of their hands or on their arms (Fig. 2b) and leaving it to dry naturally. Thus, they combine their assessment of the colors of the indigo paste in its wet and dry states to determine its quality, believing that high-quality indigo paste should appear dark-blue and purple-red when both wet and dry. In Zhenfeng County, these assessments are known as *water color* and *dry color*, respectively. Because of the inconvenience of transporting wet indigo, people dry indigo in the sun in Xianyou County of Fujian Province, and they assess the quality by observing only the color of the dried indigo blocks (Fig. 2c).

People in all the regions under study used the touch criterion to evaluate indigo quality, which involves rubbing a small amount of moist indigo paste between the index finger and thumb (Fig. 2d). Indigo paste that is smooth and difficult to wipe off is considered of high quality. Noticeable graininess indicates slightly low quality.

In Guizhou and Yunnan Provinces, local people think that high-quality indigo paste has a *sweet* (gai), *spicy* (dou), or *bitter* (æ) taste and dyes fabrics easily (Fig. 2e, f). However, notably, there were differences in the description people provided for the taste criterion and it was more common in Guizhou Province. Although more than 25% of the informants in Guizhou used this criterion, different informants offered contrasting descriptions for taste. Some informants declared that indigo paste is of good quality when it has a sweet taste, whereas a spicy or bitter taste indicates poor quality. Other informants held the opposite view.

### Quantitative evaluation of the quality criteria

The color criterion showed the highest FC value (FC = 283), QI value (QI = 1), and FL value (100%) in all study areas. In contrast to the other criteria, the color criterion was the most commonly used and recognized criterion among people (Table 3). Although the touch criterion was used in all study areas, its frequency and importance differed across regions; the highest QI and FL values were determined in Fujian Province (QI = 0.21; FL = 21%), followed by Guizhou Province (QI = 0.12; FL = 12%), while Yunnan Province showed the lowest values (QI = 0.07; FL = 7%). The taste and dyeing-ability criteria were used only in Guizhou and Yunnan Provinces, and there were differences in the frequency and importance of these two quality criteria in the two regions. In Guizhou Province, the taste criterion (FC = 47) was more frequently used than the dyeing-ability criterion (FC = 11), whereas people used the dyeing ability criterion (FC = 3) more than the taste criterion (FC = 2) in Yunnan Province, although the quantitative analysis showed a small difference in the FC value of these two criteria in this province. The QI values of the taste and dyeing-ability criteria in Guizhou Province were 0.27 and 0.06, respectively, and the FL values were 27% and 6%, respectively. However, in Yunnan Province, the QI values of the two criteria were 0.03 and 0.04, respectively, and the FL values were 3% and 4%, respectively. These findings indicated that the taste and dyeing-ability criteria were more important in Guizhou Province than those in Yunnan Province and were more frequently used by people in Guizhou Province, especially the taste criterion.

Overall, the quantitative analysis revealed that the most important and recognized evaluation criterion among informants was color. The other three criteria (taste, touch, and dyeing ability) appear to be accessory criteria.

### Verification of traditional knowledge using modern scientific methods

**Fig. 3:**
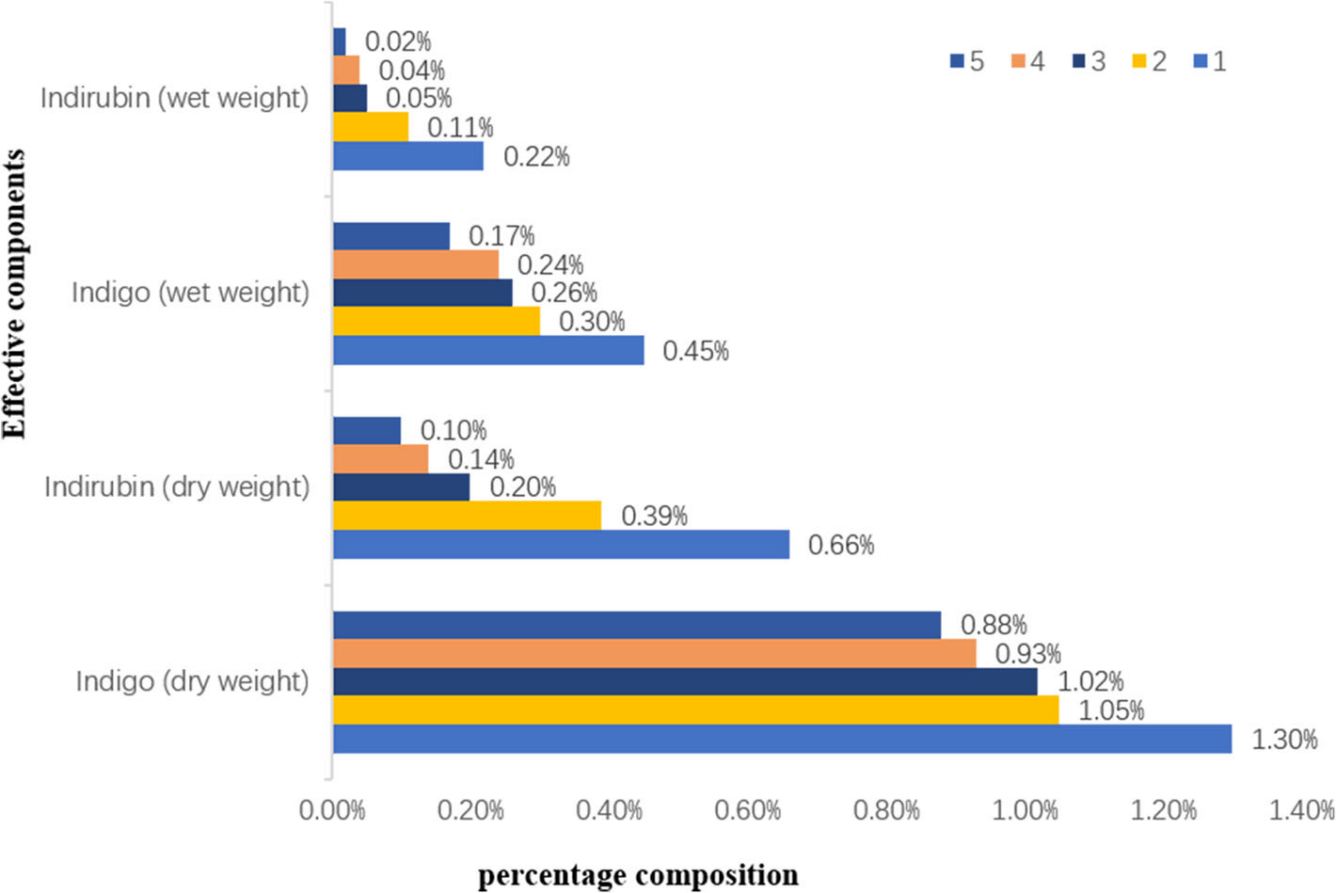
The average active ingredient content in each quality grade of indigo paste is shown where different colors represent different quality grades

The main active ingredients identified in the indigo-paste samples were indigo and indirubin. In addition, some samples contained minute amounts of indican or indole (Table 4). As the indigo paste contained water when it was sampled, we also considered water as a factor. Our findings showed that the average indigo and indirubin content decreased with a decrease in quality grade (Fig. 3). Furthermore, there was a positive correlation between the active ingredient content and quality grade of the indigo paste. One-way ANOVA showed that, whether wet or dry, there were significant differences in active ingredient content among the different quality grades.

**Table 4:** Active ingredients, pH, and particle size of indigo-paste samples from Guizhou Province

Sample number	Quality grade	Effective components content(ug/g)		Percentage of effective ingredients (%)				PH**	Particle size(d=nm)
		Indigo	Indirubin	Indigo(W) *	Indirubin(W) **	Indigo(D) **	Indirubin(D) **		
1-1	1	11268.55	10221.73	0.37	0.33	1.13	1.02	9.10	531.2–825 (100%)
1-2	1	14486.60	5441.57	0.41	0.15	1.45	0.54	9.47	396.1–825 (100%)
1-3	1	13218.67	4096.02	0.57	0.18	1.32	0.41	9.13	91.28–1281 (87.4%)/4145–5560 (12.6%)
2-1	2	10466.89	3174.17	0.21	0.06	1.05	0.32	10.03	342–825 (90.9%)/4801–5560 (9.1%)
2-2	2	10583.21	4189.60	0.41	0.16	1.06	0.42	9.18	91.28–164.2 (12.7%)/396.1–1106 (87.3%)
2-3	2	10590.91	4366.99	0.28	0.12	1.06	0.44	10.92	122.4–255 (17.2%)/615.1−2669 (82.8%)
3-1	3	10164.60	2903.67	0.23	0.07	1.02	0.29	10.98	141.8–255(19%)/531.2–1718 (81%)
3-2	3	8840.08	2357.28	0.20	0.05	0.88	0.24	10.83	220.2–712.4 (100%)
3-3	3	10672.38	2022.68	0.11	0.02	1.07	0.20	10.66	105.7–190.1 (23.6%)/531.2–1106 (76.4%)
3-4	3	10430.08	1921.20	0.32	0.06	1.04	0.19	10.21	141.8–255 (29.7%)/825–2305 (70.3%)
3-5	3	10832.98	1193.15	0.46	0.05	1.08	0.12	11.55	220.2–955.4 (96%)/4145–5560 (4%)
3-6	3	10457.34	2065.58	0.26	0.05	1.05	0.21	10.85	91.28–164.2 (17.4%)/342–825 (82.6%)
3-7	3	10097.03	1470.28	0.25	0.04	1.01	0.15	11.29	78.82–141.8 (13.2%)/220.2–955.4 (86.8%)
4-1	4	7976.97	1818.84	0.16	0.04	0.80	0.18	11.06	91.28–141.8 (8.5%)/458.7–1106 (88.9%)/5560 (2.7%)
4-2	4	9235.30	1134.66	0.37	0.05	0.92	0.11	10.39	122.4–220.2 (21.5%)/531.2–1718 (76.6%)/5560 (1.9%)
4-3	4	10334.90	1386.50	0.18	0.02	1.03	0.14	11.36	122.4–220.2 (30.1%)/531.2–1281 (67.2%)/5560 (2.8%)
4-4	4	9251.13	1480.47	0.20	0.03	0.93	0.15	11.34	78.82–164.2 (13.2%)/255–712.4 (86.8%)
4-5	4	9691.15	1316.95	0.29	0.04	0.97	0.13	11.53	91.28–825 (84.4%)/3580–5560 (15.6%)
5-1	5	7834.44	1103.13	0.10	0.01	0.78	0.11	11.22	78.82–105.7 (7.4%)/396.1–955.4 (92.6%)
5-2	5	8106.15	1062.89	0.14	0.02	0.81	0.11	11.64	91.28–141.8 (7.5%)/295.3–825 (80%)/4145–5560 (12.5%)
5-3	5	10369.98	894.61	0.26	0.02	1.04	0.09	11.43	141.8–295.3 (24.9%)/712.4–1484 (75.1%)

Significance is indicated by *P < 0.05, **P < 0.001

Note: *W* wet weight, *D* dry weight

Regarding the pH of the 21 indigo-paste samples, the minimum, maximum, and average values were 9.10, 11.64, and 10.67, respectively (Table 4). We identified a relationship between pH and quality grade; the low-quality indigo-paste samples tended to have high pH values and vice versa. One-way ANOVA showed that there were significant differences in indigo paste pH among different quality grades (*P* = 0.000). Within a certain range (9 ≤ pH ≤ 12), the pH value of the high-quality indigo paste was relatively low, whereas the pH value of the poor-quality indigo paste was relatively high.

**Fig. 4:**
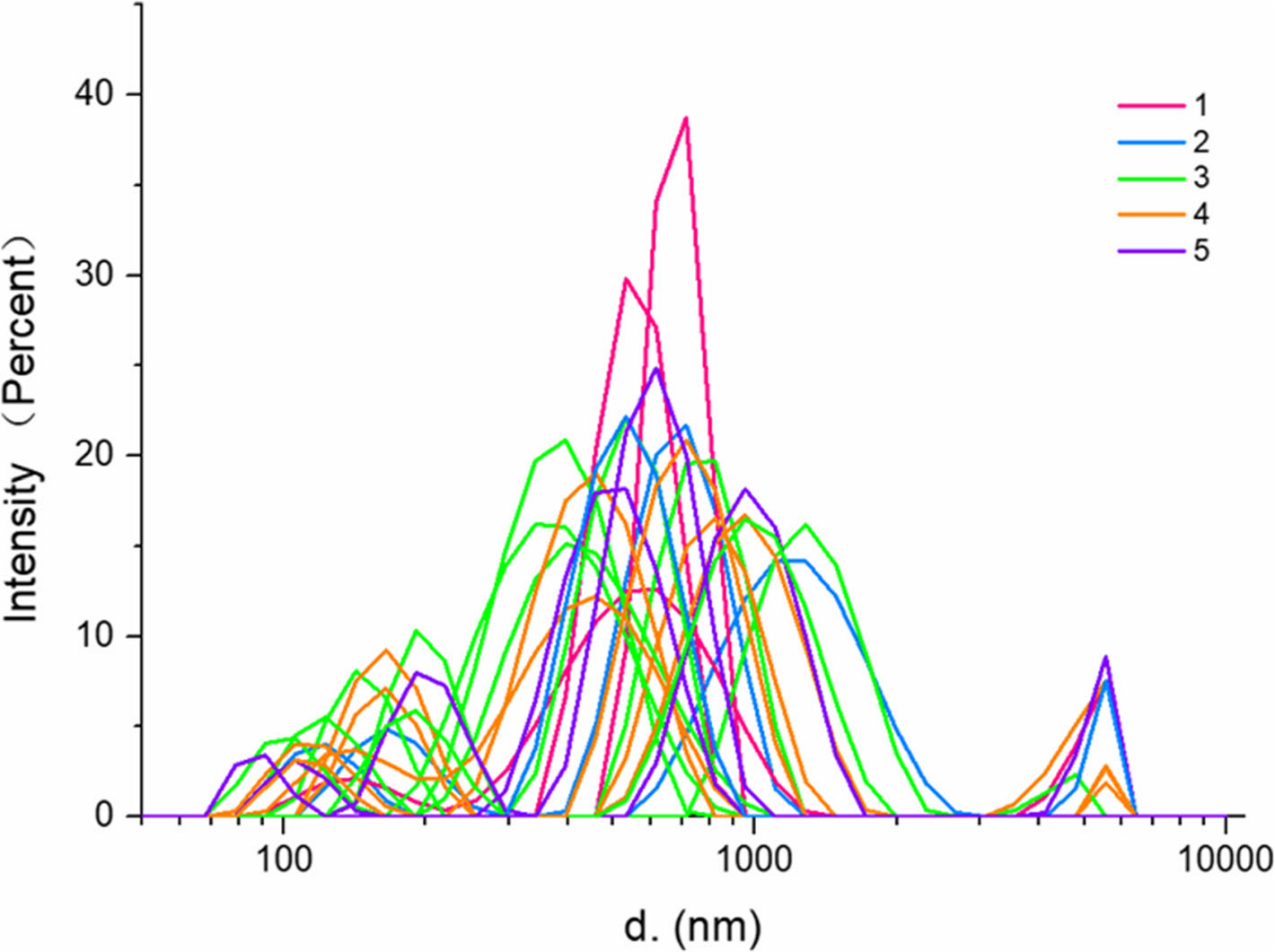
A broken line graph of the particle size distribution of 21 indigo-paste samples. Different quality grades are indicated by different colors

The particle size distribution of the indigo-paste samples ranged from 78.82 to 5560 nm, with the particle size of most samples ranging between 200 and 2600 nm (Table 4). All samples had two or three distribution intervals, except for three samples (1-1,1-2,3-2), which were distributed at continuous intervals. As indicated in Fig. 4, the indigo-paste samples in each quality grade did not have an obvious independent distribution interval and were randomly distributed across the particle size range. Evidently, there was no correlation between quality grade and particle size.

## Discussion

### Sociocultural characteristics of indigo-paste artisans

We documented the traditional knowledge and experience of 283 informants to analyze how they assessed the quality of indigo paste. As a commodity, indigo paste plays different roles in different regions. In Zhenfeng County of Guizhou Province and Xianyou County of Fujian Province, the indigo-paste trade provides the main source of income for the local Han farmers. In contrast, in Congjiang County of Guizhou Province, the indigo-paste trade serves as a traditional model of national self-sufficiency. Both these models exist in Yuanyang and Jinping Counties of Yunnan Province.

#### Division of labor: sex of indigo-paste artisans

The different social roles of indigo paste lead to different social divisions of labor. With regard to the sex of indigo-paste artisans, there were more men (81.3%) engaged in indigo-paste production than women (18.7%) in Zhenfeng County of Guizhou Province as well as in Xianyou County of Fujian Province (71.4% men versus 28.6% women). However, in Congjiang County in Guizhou Province, only women were engaged in the production of indigo paste, following the traditional self-sufficiency model (Table 2). Although both production models exist in Yunnan Province, at the time of our survey, the scope and quantity of the trade was relatively small and based mainly on the traditional model of national self-sufficiency; consequently, the number of women (88.6%) engaged in indigo-paste production was almost eight times the number of men (11.4%). These results are consistent with the findings of previous studies, which reported that traditional dyeing knowledge is transmitted matrilineally, and dyeing is mainly mastered and performed by women [45, 46]. Furthermore, activities such as dyeing are considered inappropriate for men [47]. However, when indigo paste becomes a tradable commodity and generates economic benefits, men readily become involved in this work [46]; indeed, they may even participate as the main labor force.

#### Division of labor: age of indigo-paste artisans

Regarding the age of the indigo-paste artisans, in Congjiang County, mainly young women aged 30–49 years (50.4%) were engaged in the production and use of indigo paste; 40.3% were 50–69 years old, and only 9.3% were over 70 years old. In the local area, making indigo paste and dyeing cloth seemed to be the daily work of minority women. In other regions, the indigo-paste artisans were mainly elderly women aged 50–69 years (68.8% in Zhenfeng, 54.3% in Yunnan, and 76.2% in Fujian), whereas young women aged 30–49 years were relatively rarely involved in indigo-paste production (28.1% in Zhenfeng, 25.7% in Yunnan, and 9.5% in Fujian). Overall, young people aged 30–49 years (35.7%) and middle-aged and elderly people aged 50–69 years (52.3%) showed extensive knowledge and artisanship of indigo-paste production. However, previous studies indicate that the majority of traditional knowledge is usually held by the elderly, whereas young women do not prove to be very knowledgeable about the trade [12, 48].

### Characteristics and material basis of traditional knowledge about indigo paste

#### Color and active ingredients

**Fig. 5:**
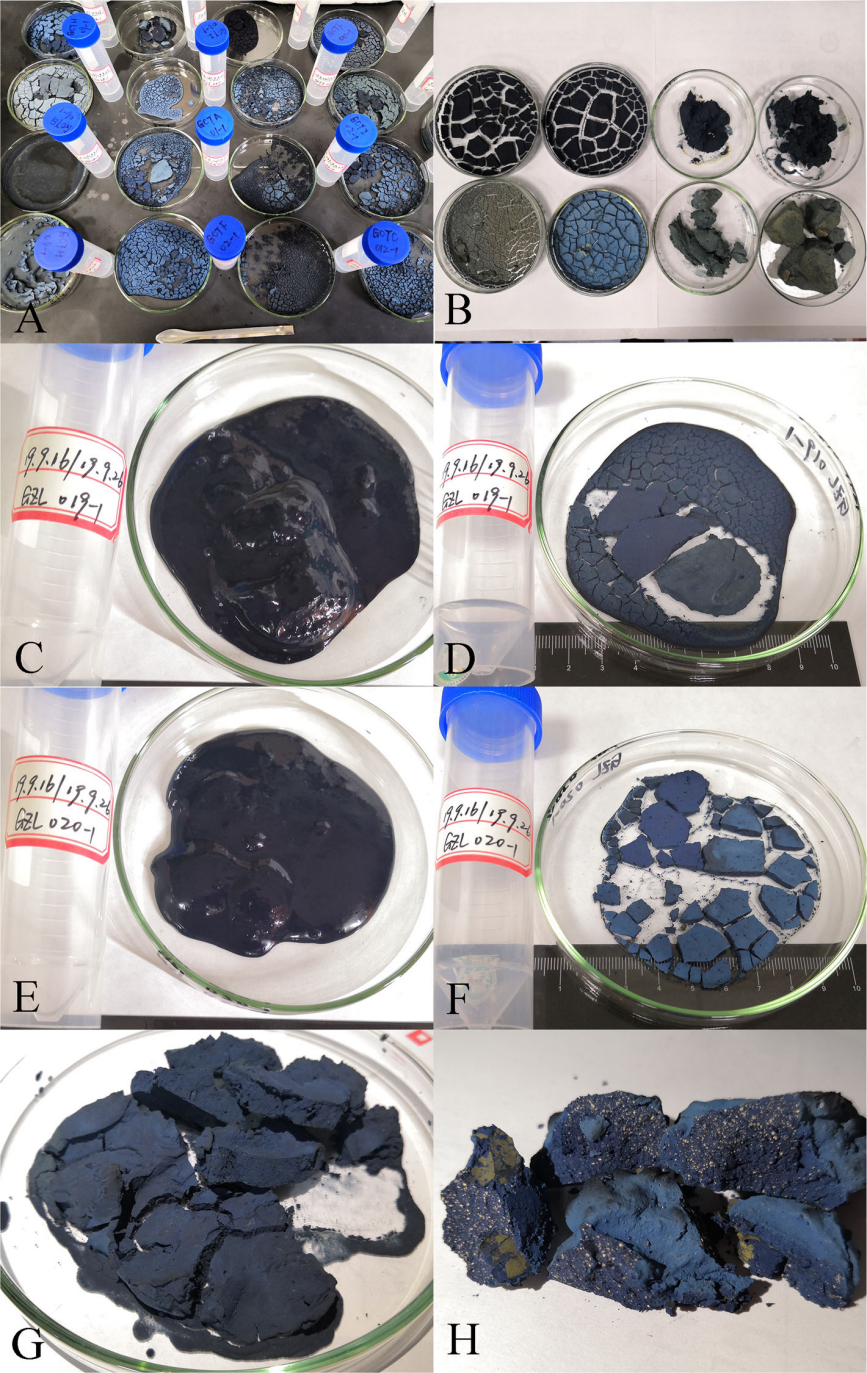
The status of indigo paste of different quality grades after drying. **a**, **b**, **d**, **f** Different colors of the indigo paste after drying. **g**–**h** After drying, the lime particles and impurities in indigo paste can be observed.

By drying the indigo-paste samples in the laboratory, we observed that there were no significant differences in color or gloss of indigo paste in its wet state, except in the very high- and very low-quality samples. However, color differences were noticeable after drying (Fig. 5). This indicates a degree of rationality associated with the simultaneous observation of water color and dry color among the people of Zhenfeng County, in addition to the importance of color in assessing indigo-paste quality. Our survey revealed that locals believe a high-quality indigo paste should have dark-blue hue and purple-red metallic luster. Generally, the blue hue in indigo paste is due to its indigo content, whereas its indirubin content produces the purple-red luster [49]. Thus, the ratio of the indigo to indirubin content determines the color of the indigo paste. The HPLC-DAD quantitative analysis confirmed that the quality of indigo paste was related to the content of indigo and indirubin. The higher the content of indigo and indirubin—especially indirubin—the better is the quality of the indigo paste. All these findings confirmed that the color of the indigo paste was an adequate quality criterion. However, due to the limited number of experimental samples, the range of indigo and indirubin contents as well as the color distribution range of the different quality grades of indigo paste could not be identified in this study. Determining these ranges requires further research.

#### Taste and pH

**Fig. 6:**
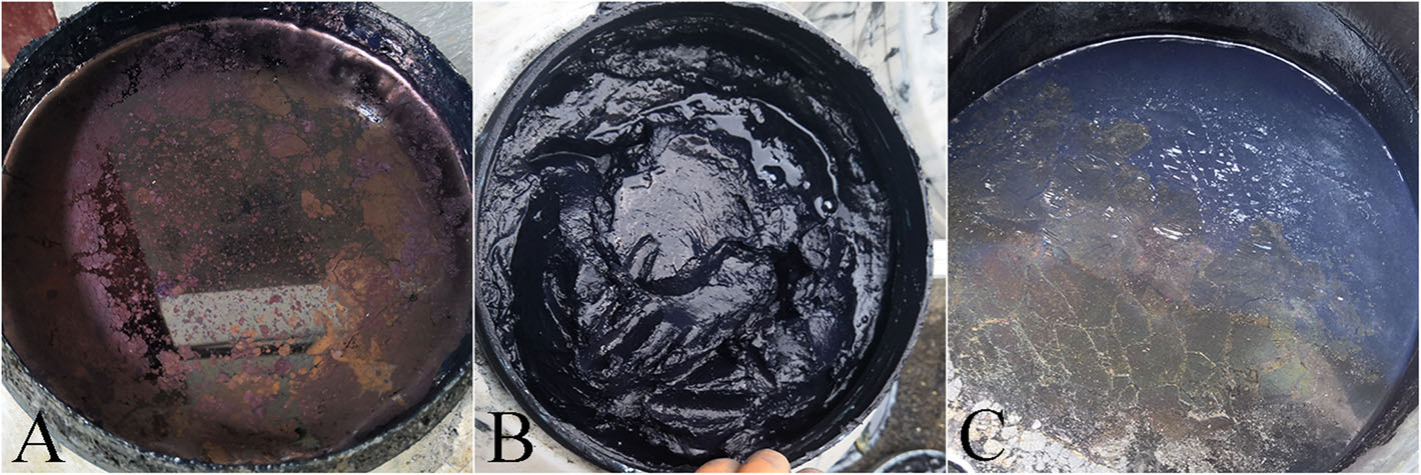
Different colors of the wetting indigo paste

In addition to taste variation among indigo-yielding plants, the taste of indigo paste is strongly related to the amount of lime added during the production process, where adding too much or too little lime results in a low-quality indigo paste. As different alkalinity stimulates human taste buds differently, local people evaluate whether the correct amount of lime has been added based on a simple taste sensation, thus judging the quality of the indigo paste. However, our survey data revealed that the taste descriptions provided by the informants differed and were even contradictory. There are two possible reasons for these differences: one is that taste description is mainly influenced by personal subjectivity, and the other is that informants have different perceptions and descriptions of taste due to cultural divergence. This phenomenon also occurred with respect to the use of the color criterion. For example, some people might describe high-quality indigo paste as being red in color, despite an apparent purplish-red luster (Fig. 6).

#### Touch and particle size

**Fig. 7:**
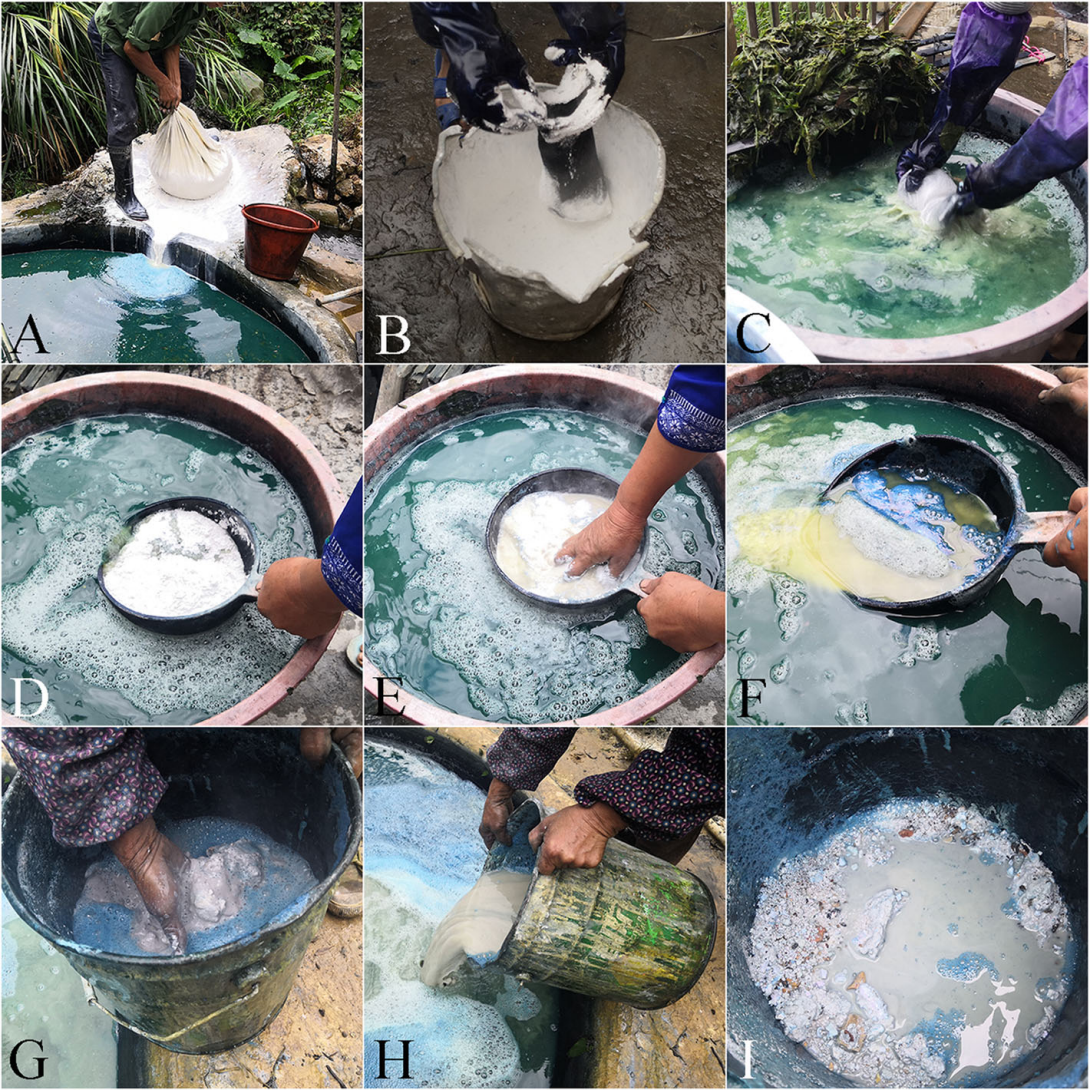
The way in which lime is added in the process of indigo-paste production. **a**–**c** put lime into a cloth or gauze bag. **d**–**i** Put lime in a water scoop or bucket

The particle size quantitative analysis confirmed that there was no correlation between quality grade and particle size. However, after drying different indigo pastes, we noted differences in appearance of the inner parts of the blocks. Some indigo-paste blocks had a uniform internal color; no lime particles or impurities were observed. In contrast, there were varying amounts of white or other colored particles in other indigo-paste blocks (Fig. 5). This observation may be related to the way in which lime is added in the process of indigo-paste production. Some informants described how they place lime in a cloth or gauze bag before adding it. They then rub the bag in the soaking liquid to produce a fine lime slurry that flows out of the bag (Fig. 7a–c). This method results in very minute lime particles and a markedly reduced number of impurities in the lime slurry. However, other informants described how they place the lime in a water scoop or bucket, add a small amount of soaking liquid, mix, and then pour this directly into the soaking solution (Fig. 7d–i). This approach ultimately results in the inclusion of large lime particles and impurities in the indigo paste. Given these different approaches to adding lime, and their potential impact on the final product, the touch criterion is necessary for the assessment of indigo-paste quality. The results of the particle size analysis may have been influenced by the large particles in the indigo paste settling in the instrument and consequently not being recorded. This potential problem needs to be resolved in future research on indigo paste properties.

### Cultural standards and industrial standards of indigo dyestuff

The chemical industry standard of the People’s Republic of China stipulates five criteria used to judge the quality of synthetic indigo. These are as follows: appearance (dark blue uniform powder or granule), mass fraction of indigo (≥ 93%), mass fraction of moisture (≤ 1.0%), mass fraction of fineness (≤ 5.0% beyond 250 μm), residue in the sieve (≤ 5.0%), and iron content (≤ 500 mg/kg). Although the target objects are different, the industrial standards of appearance, mass fraction of indigo, and mass fraction of fineness were similar to the color and touch criteria in the cultural standards. These industrial and cultural standards are all regulations based on color, active ingredient contents, and particle size. However, as organoleptic selection criteria, the folk quality criteria for judging indigo paste have a unique cultural imprint.

For the indigenous people, sensory evaluation based on odor, taste, sight, and touch plays a critical role in plant classification and traditional medicine therapy [50–53]. Such traditional knowledge is a summary of the understanding of local people about their surroundings [54]. Such knowledge may be independent of modern scientific understanding and not rely on modern scientific testing methods. For example, our survey indicated that local people preferred purple-red indigo paste to pure blue indigo paste, suggesting that in application of the cultural quality criteria, indirubin was judged to be more important than indigo. However, in the context of industrial dyestuff production, indirubin has always been regarded as a by-product [55, 56]. This difference may be related to cultural differences in the same way that links between taste perceptions and medicinal uses of herbal drugs can be markedly different across diverse cultures [57]. The color criterion we documented corresponds with the reports of indigo paste quality assessment in ancient Chinese books. For example, there is a document in *Liping Fuzhi*(Guizhou) that contains the following statement: “投入生石灰, 则满地颜色皆收入灰内, 以带紫色者为上”, which implies that purple indigo paste is the best. Similarly, there is a description in *Dyeing Sutra*: “明兰宝翠, 兹浆鲜红, 至次年春夏可变成熟红如天青缎” [58], which implies that the best quality indigo-paste should have a dark blue and red luster. In addition, the Hainan Li and Miao people think that a dark blue and reddish indigo paste is of a better quality [48]. The Yao, Zhuang, Dong, and other ethnic minorities like to dye their fabrics dark blue or black with a red color [59, 60], and the presence of indirubin can satisfy such preferences. The medicinal benefits of indirubin help to explain why people in Xianyou County favor the existence of this substance. Therefore, in a cultural context, indirubin in indigo paste is not considered a by-product but a critical determinant of indigo-paste quality.

## Conclusions

Although modernization and urbanization continue to change the traditional ways in which people produce goods, some local people still maintain the traditional culture and methods used for indigo extraction and indigo-paste preparation. This study documented four folk criteria and five quality grades of indigo paste and revealed the importance of indirubin and pH for assessing the quality through quantitative analyses. Even after thousands of years, the ancient methods used by the local people for identifying natural indigo remain comprehensive and unique. The traditional method for indigo-paste quality assessment is seemingly backward, but it is advantageous not only in its simplicity and ease of use but also in its environment-friendliness and high energy efficiency. Simple traditional knowledge can also inspire the development of modern industrial technology, possibly the invention of modern detection equipment and the exploitation of novel blue dyes. Traditional knowledge remains an invaluable cultural heritage of humanity that we need to actively preserve and transmit to new generations.

Acknowledgements

We are most grateful to all interviewee for their hospitality and willingness to share their traditional knowledge with us. We thank Professor Wenyun Chen, Yu Zhang, Yi Gou, and Ruyan Fan, for their assistance.

## Data Availability

The datasets used and/or analyzed during the current study are available from the corresponding author on reasonable request.

## Abbreviations

FC: Frequency of citation
QI: Mention Index
FL: Fidelity level
